# Genetic dissection of independent and cooperative transcriptional activation by the LysR-type activator ThnR at close divergent promoters

**DOI:** 10.1038/srep24538

**Published:** 2016-04-18

**Authors:** Elena Rivas-Marín, Belén Floriano, Eduardo Santero

**Affiliations:** 1 Centro Andaluz de Biología del Desarrollo, Universidad Pablo de Olavide/Consejo Superior de Investigaciones Científicas/Junta de Andalucía, and Departamento de Biología Molecular e Ingeniería Bioquímica, Universidad Pablo de Olavide, Spain.

## Abstract

Regulation of tetralin biodegradation operons is one of the examples of unconventional LysR-type mediated transcriptional regulation. ThnR activates transcription from two divergent and closely located promoters P_*B*_ and P_*C*_. Although ThnR activates each promoter independently, transcription from each one increases when both promoters are together. Mutational analysis of the intergenic region shows that cooperative transcription is achieved through formation of a ThnR complex when bound to its respective sites at each promoter, via formation of a DNA loop. Mutations also defined ThnR contact sites that are important for independent transcriptional activation at each promoter. A mutation at the P_*B*_ promoter region, which abolishes its independent transcription, does not affect at all P_*B*_ transcription in the presence of the divergent promoter P_*C*_, thus indicating that the complex formed via DNA loop can compensate for the deficiencies in the correct protein-DNA interaction at one of the promoters. Combination of mutations in both promoters identifies a region at P_*C*_ that is not important for its independent transcription but it is essential for cooperative transcription from both promoters. This work provides new insights into the diversity and complexity of activation mechanisms used by the most abundant type of bacterial transcriptional regulators.

LysR-type transcriptional regulators (LTTRs) are the most abundant family of bacterial regulators, also being found in archaea^1^,^2^,^3^ and algal chloroplasts^4^. They play an essential role in the control of very important processes such as the utilisation of many different carbon sources, including organic contaminants^5^, responses to oxidative stress, secondary metabolite production, motility, biofilm formation or virulence/pathogenesis among others^6^.

The most common arrangement for an LTTR-regulated system consists of two divergent operons encoding the LTTR and the target genes, respectively. Most LTTRs act as transcriptional activators of their target genes whilst transcriptionally repressing their own expression. To do so, the LTTR binds to a high affinity palindromic binding site, containing a conserved T-N_11_-A motif, centred near position −65 relative to the target operon transcription start site and overlapping the LTTR-coding gene divergent promoter. This primary binding site (PBS) (also denoted RBS in other systems for Repression Binding Site) is essential for the LTTR binding and for both negative autoregulation and activation of the target promoter. LTTRs are usually tetramers and, upon binding to the PBS, make additional contacts with non-conserved sequences that extend downstream of the −35 box of the target activated promoters. This secondary binding site (SBS) (also known as ABS in other systems for Activation Binding Site), is essential for activation. LTTRs typically interact with their binding sites in the absence of the inducer signal but, upon binding the inducer molecule, they suffer a conformational change, detectable in many instances by qualitative alterations in protein-DNA interactions and/or DNA topology, which is critical for activation (reviewed by Díaz and Prieto^7^; Tropel and van der Meer^5^).

However, there are increasing examples of LTTR-regulated systems that do not fit in this canonical arrangement. There are some regulators that activate multiple promoters^8^,^9^ and others that can activate several target promoters while repressing others^10^,^11^. Some LTTRs require co-regulators to detect and transduce the signal^12^,^13^,^14^. Regarding promoters, some contain multiple binding sites for the regulator^15^, whilst others contain binding sites for the LTTRs and for additional co-regulators^16^,^17^,^18^,^19^. These data indicate that LTTRs do not share a unified mechanism of activation.

One of such unconventional systems is that regulating the biodegradation of the organic solvent tetralin in *Sphingopyxis granuli* (formerly *Sphingopyxis macrogoltabida*) strain TFA. The genes coding for the degradation pathway are grouped in four operons (Fig. 1a) induced in response to tetralin and subjected to catabolite repression^20^,^21^. ThnR is an LTTR essential for transcription activation from the four promoters P_*C*_, P_*B*_, P_*H*_ and P_*M*_^22^,^21^. Expression of *thn* operons also requires ThnY^23^, a ferredoxin reductase-like protein that has evolved to function as a co-activator of the *thn* operons^24^, which mediates a unique modulation system that prevents gratuitous induction of the *thn* operons by molecules that, being similar to tetralin, are not substrates of the pathway^14^. ThnY slightly increases binding affinity of ThnR to the promoter regions but it does not bind DNA by itself and does not alter the footprint pattern generated by ThnR at these regions. Rather, it co-activates transcription by activating ThnR via protein-protein interactions^24^. The regulatory proteins are expressed from both a constitutive promoter (P_*R*_) and the tetralin inducible P_*C*_.

Two of the promoters, P_*B*_ and P_*C*_, are divergently transcribed and closely located. Each promoter region bears a primary binding site (PBS) (Fig. 1b), which is also conserved in the other ThnR-activated promoters. Footprinting analyses indicated that ThnR binds to these PBS but also makes additional contacts with DNA sequences located downstream, which extended to the promoters^22^ (these regions are denoted as SBS in Fig. 1b). Mutations of the PBSs showed that they are essential for transcription from their cognate promoters, thus assigning them a functional role in *thn* transcription activation, while also affecting the transcription from the divergent promoters, indicating that transcription from both promoters was coordinated^22^.

This work reports on the results of a more exhaustive mutational analysis of this divergent promoters region, assigns new functions to DNA sequences between the PBSs and their cognate promoters, provides further insights into the mechanism of coordinated activation of these divergent promoters and confirms the formation of a complex when ThnR is bound to their distant sites via a DNA loop. Furthermore, the results reveal an additional function of a DNA loop not uncovered so far: compensation of non-productive protein-DNA interactions at an activating site.

## Results

### Cooperative and independent transcriptional activation of the P_*B*_ and P_*C*_ promoters

**Figure 1:**
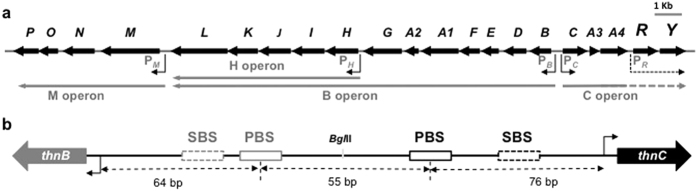
Transcriptional arrangement of the *thn* operons with all detected operons (**a**) and a schematic zoom of the intergenic *thnBC* region showing the ThnR primary and secondary binding sites at each promoter region, and the BglII restriction site used for isolation of each promoter region for independent transcription analysis (**b**).

To analyse expression levels of independent and cooperative transcriptional activation from each divergent promoter, two types of gene fusions were used. For cooperative activation analysis, translational fusions were constructed to each *thnB* or *thnC* gene using a DNA fragment that bears both promoters and the whole intergenic region. Independent transcription was estimated by using shorter promoter fragments containing just one of the promoter regulatory regions, just up to the BglII site separating the promoter regions (Fig. 1b). The gene fusions were integrated into the chromosome of the strain T-690, in which a 12.2 kb DNA region covering the whole *thnC* operon and the *thnB* operon down to *thnG* was substituted by a Kanamycin resistance cassette (Fig. 1). The gene fusions were integrated in the same genetic context as the *thn* operons by a single recombination event between the Km^R^ genes present in both the plasmid and the chromosome, as previously described^22^. Since T-690 lacks the transcriptional activators ThnR and ThnY, the *thnRY* operon transcribed from its own constitutive promoter was provided in the low copy number vector pLAFR3 (pMPO943).

**Figure 2:**
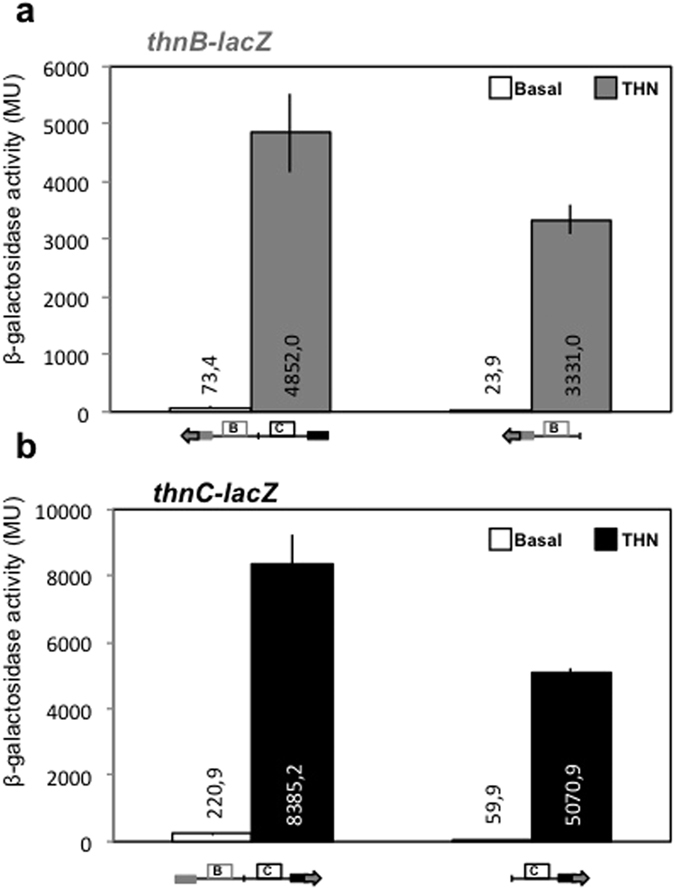
Cooperative and independent transcription activation from P_*B*_ (**a**) and P_*C*_ (**b**) promoters, using the large and the short *lacZ* gene fusions to each promoter. Boxes represent the promoter regions. The arrows downstream of some of the promoters represent the *lacZ* gene fusions to these promoters. Basal and tetralin-induced (THN) expression levels are shown.

These *thn::lacZ* gene fusions and others that bore substitutions in each primary binding site B and C were previously constructed and their effects on transcription from their cognate or the divergent promoters when ThnR and ThnY were highly expressed from the heterologous P*tac* promoter, were reported^22^. The results of similar experiments but producing the activators from their own promoter in a low copy number plasmid are shown in Fig. 2. Low expression of both ThnR and ThnY in T-690 is enough to induce *thn* genes expression in the presence of tetralin. Independent expression from each promoter, i. e. in the absence of its divergent partner, was obvious after induction with tetralin. Expression from P_*B*_ was lower than that from P_*C*_, as previously described^19^. However, expression levels from both promoters increased when the divergent promoter was also present (146% and 165% for P_*B*_ and P_*C*_, respectively).

In this new system, expression from P_*B*_ did not respond as previously described, when expression of the activators was high, in which induced P_*B*_ expression was actually higher when isolated from P_*C*_^22^. In the previous system the regulatory *thnRY* operon was transcribed from the strong P*tac* promoter in a medium copy number plasmid; therefore, the amount of regulators was presumably quite high. However, in the system used here ThnR and ThnY are produced using their own P_*R*_ promoter, in a low copy number plasmid, thus presumably producing an amount of the regulators closer to the wild-type situation. The expression levels from P_*B*_ and P_*C*_ in this system are more similar to those obtained in the wild type strain bearing the *lacZ* fusions integrated in the chromosome^19^ than those shown when *thnRY* were transcribed from the P*tac* promoter, which were significantly higher^22^.

### Transcriptional coupling and face of the helix dependence for cooperative transcription

Expression of many *E. coli* genes is sensitive to supercoiling^25^ and it is known that an elongating RNA Polymerase-RNA complex may cause a change in local DNA superhelicity^26^. Therefore, at close divergent promoters, transcription from one promoter may affect transcription levels from the other, as it has been described for the divergent *ilvY* and *ilvC* promoters regulated by the LTTR IlvY^27^,^28^. One possible mechanism for P_*C*_ and P_*B*_ cooperation is that local transcription from the neighbour promoter results in supercoiled structure changes on second promoter, thus mutually benefitting from this coupling. Alternatively, cooperation may involve positive protein-protein interactions between the ThnR activator molecules bound at each promoter region.

**Figure 3:**
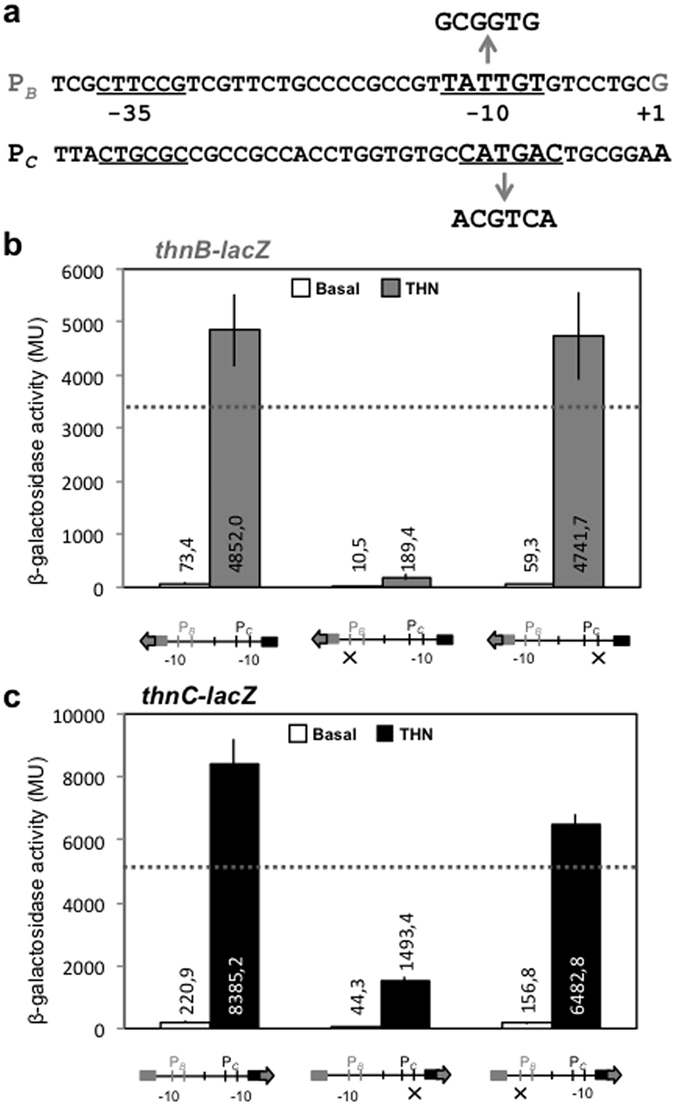
Effect of mutations at the −10 regions of P_*C*_ and P_*B*_. (**a**) Intergenic *thnB-C* region indicating the −10 and −35 promoter regions of each promoter and the constructed mutations. (**b**) Expression levels of *thnB-lacZ* gene fusions. (**c**) Expression levels of *thnC::lacZ* gene fusions. The horizontal dotted lines represent the independent levels of expression (short *lacZ* gene fusion).

Mutations of the putative −10 box of each promoter were constructed (Fig. 3a), and its effect on transcription form its own and divergent promoters, was tested. As shown in Fig. 3b,c, mutations of the −10 boxes severely reduced the expression from their respective promoter, thus showing their importance as part of each promoter. Mutation of the −10 box of P_*C*_ did not modify induced P_*B*_ expression levels at all (Fig. 3b), thus indicating that the cooperative effect in P_*B*_ transcription does not require transcription from P_*C*_. On the other hand, P_*C*_ expression was slightly affected in a negative way by the mutation in the P_*B*_ −10 box (Fig. 3c). However, importantly, cooperation between the two promoters was detected even in the presence of the P_*B*_ −10 box mutation, as the expression levels driven by this construct were higher than those of carrying the independent P_*B*_ or P_*C*_ promoters (dotted lines in Fig. 3b,c).

**Figure 4:**
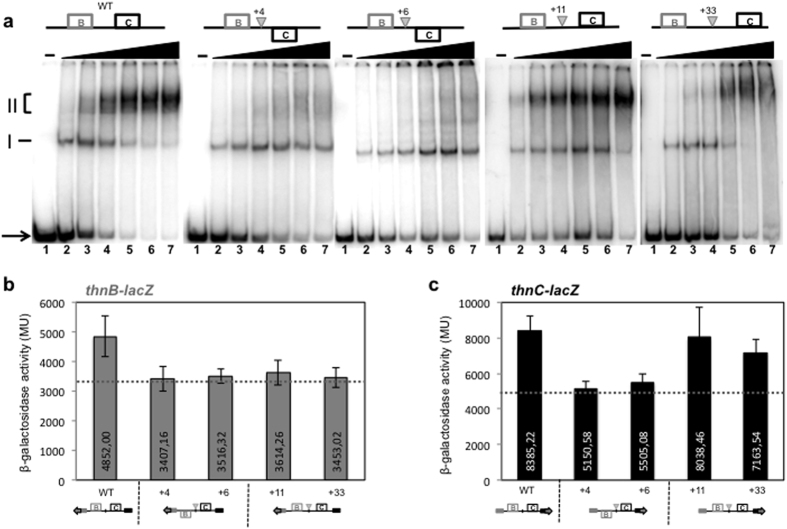
(**a**) EMSA assays showing complex I and complex II formation at the *thnB-thnC* divergent promoter region in WT and +4, +6, +11 and +33 bp insertion mutants. (**b**) Effects of the insertions on *thnB* expression. (**c**) Effects of the insertions on *thnC* expression. The horizontal dotted line represents independent levels of expression. Basal levels of the *thnB::lacZ* gene fusions ranged between 73 and 137 MU and those for the *thnC::lacZ* gene fusions ranged between 127 and 364 MU. The increasing concentrations of ThnR tetramers are: 0, 25, 50, 75, 100, 125 and 150 nM.

The centres of the ThnR primary binding sites for each promoter are separated by 55 bp, corresponding to 5 integral turns of the helix (Fig. 1b), thus both sites are oriented on the same face of the helix. In order to test the effect of further separating these bindings sites or placing them on opposite faces of the helix, +4, +6, +11 and +33 bp were inserted between the binding sites (Fig. 4). These modified promoter regions were used as probes to analyse DNA-protein interactions and to construct *thnB* and *thnC* gene fusions to *lacZ*, which were inserted into the chromosome of the strain T-690 to characterise their effect on transcription. As previously reported^22^, ThnR initially binds to its highest affinity PBS close to *thnC* (complex I) but at higher concentrations ThnR binding resulted in the formation of a complex II due to protein-protein interaction between ThnR bound to the sites of each promoter region (Fig. 4a). Insertion of half turns of the helix (+4 and +6 bp) did not affect initial binding but had a dramatic effect on formation of the complex II structure, as evidenced by electrophoretic mobility shift assays (EMSA) (Fig. 4a), whilst insertion of integral turns of the helix (+11 and +33 bp) still allowed it. Footprinting analyses showed that ThnR can bind to both sites B and C in the outphased promoters but they lack the hypersensitive band shown by the wild-type DNA region at position −103 from the P_*C*_ transcriptional start, between the primary binding sites B and C, which is indicative of a DNA distortion. This band still remains in the +11 and +33 bp mutants (Supplementary Fig. S1). These data strongly suggest that two ThnR molecules bound to their respective sites in the intergenic region form a complex through ThnR protein-protein interactions that results in formation of a DNA loop, which cannot be formed if the ThnR primary binding sites are not aligned in the same face of the helix. These mutant regions were used to construct *lacZ* gene fusions, and were integrated into the chromosome of T-690 as described above, to analyse expression from each promoter. Insertion of half turns of the helix reduced expression from both promoters down to almost the levels of independent transcription (Fig. 4b,c). On the other hand, insertion of integral turns of the helix allowing formation of the complex did not substantially affect the *thnC* expression levels (Fig. 4c), thus suggesting that cooperative transcription from P_*C*_ relies on ThnR interactions when bound to distant sites with the consequent formation of the DNA loop. Interestingly, expression from P_*B*_ was also reduced to independent levels of expression when the binding sites were separated by 1 or 3 turns of the helix (Fig. 4b), thus indicating that cooperative transcription of *thnB* is highly sensitive to the distance between the primary binding sites.

### Cis-acting sequences at the P_C_ promoter region relevant for transcription

**Figure 5:**
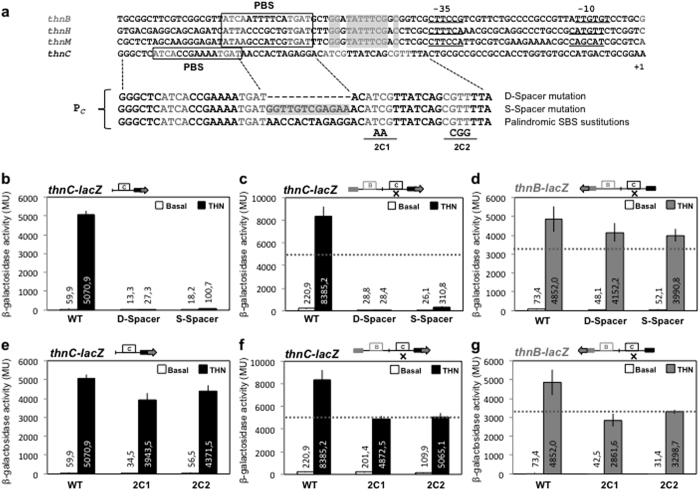
(**a**) Alignment of the *thn* promoters showing the difference between P_*C*_ and the other promoters, and mutations constructed in the P_*C*_ promoter region. Effect of each spacer mutation on independent transcription from P_*C*_ (**b**) or on coordinated transcription from P_*C*_ (**c**) and P_*B*_ (**d**). Effect of the SBS substitutions on independent transcription from P_*C*_ (**e**) or on coordinated transcription from P_*C*_ (**f**) and P_*B*_ (**g**). The horizontal dotted lines represent the independent levels of expression.

The arrangement of ThnR binding sites at the P_*B*_, P_*H*_ and P_*M*_ promoter regions is conventional whilst at the P_*C*_ promoter region is unusual (Figs 1b and 5a). First, its PBS, which is essential for ThnR binding and ThnR-mediated activation of P_*C*_, is centred at −76, one helix turn further upstream of the transcription start site compared to the conventional arrangement. Secondly, a palindromic motif resembling the PBS is found 29 bp downstream of the primary site, which is also protected from DNase I digestion by ThnR binding^22^ (Supplementary Fig. S1). This SBS, though located in the same position relative to the transcription start site as the conserved regions in the P_*B*_, P_*H*_ and P_*M*_ promoters, does not show similarity to the conserved sequence found in them (Fig. 5a).

To characterise the sequences required for transcriptional activation of P_*C*_, mutations affecting the palindromic SBS and the spacer between both binding sites were made and used to construct *lacZ* gene fusions, which were integrated in T-690, as described above. The D-Spacer mutation is a 12 bp deletion that places the primary binding site one turn of the helix closer to the promoter, in the canonical position found in other ThnR and LysR-type activated promoters. The S-Spacer mutation consists of transition mutations at each of the first 12 bp of the spacer region.

As shown in Fig. 5b, both the deletion and the substitution of the spacer virtually abolished independent transcription from P_*C*_. Therefore, presence and identity of the spacer sequences are essential for P_*C*_ transcription. In the complete context comprising both promoters the effect of both mutations on transcription from P_*C*_ was identical, thus indicating that function of the spacer sequence cannot be compensated at all by the cooperative interaction with the ThnR bound at site B (Fig. 5c). Although virtually unable to promote transcription from P_*C*_, the mutated spacer still allowed complex II formation (Supplementary Fig. S2), thus suggesting that ThnR still binds to this region although contacts are not productive for P_*C*_ transcription. Both spacer mutations *in site* C had a clear effect on transcription from the divergent P_*B*_ promoter but expression levels were higher than those of independent expression (Fig. 5d), thus cooperative expression from P_*B*_ was still detectable.

Mutations S1 to S5 are substitutions of 3 bases spanning the 12 bp spacer sequence. With the exception of S1, which did not reduce P_*C*_ expression, different substitutions of 3 nucleotides spanning the 12 bp spacer sequence had similar partial defects in P_*C*_ transcription, thus indicating that the first 10 nucleotides of the spacer are similarly important for function (Supplementary Fig. S3).

Mutations 2C1 and 2C2 are nucleotide substitutions at the upstream or the downstream half of the palindromic secondary site, respectively (Fig. 5a). The effect of each mutation on independent expression from P_*C*_ was similarly weak as *thnC::lacZ* gene fusions bearing these mutations showed 78% and 86% of the wild-type levels, respectively (Fig. 5e). This weak effect clearly indicates that the secondary palindromic site is not important for independent transcription from P_*C*_, which is surprising given the sequence similarity to the primary site and the fact that ThnR also protected this site from DNase I digestion^22^ (Supplementary Fig. S1). However, their effects on transcription from P_*C*_ were greater when analysing the effects of these mutations in a complete context bearing both promoter regions, as expression was reduced down to 58% and 60%, respectively, which are equivalent to the independent expression levels obtained with the isolated wild-type P_*C*_ promoter (Fig. 5f). Footprinting analysis of the whole promoter region bearing the 2C mutations (Supplementary Fig. S4) showed that ThnR did not protect or protected weakly the region corresponding to the palindromic SBS in the mutants. Similarly evident effects were also observed on cooperative transcription from the divergent P_*B*_ promoter, which showed expression levels similar to the independent expression from the isolated P_*B*_ wild-type promoter (Fig. 5g), thus suggesting that this palindromic SBS in P_*C*_ is important for cooperative transcription from both promoters.

### Cis-acting sequences at the P_B_ promoter region relevant for transcription

**Figure 6:**
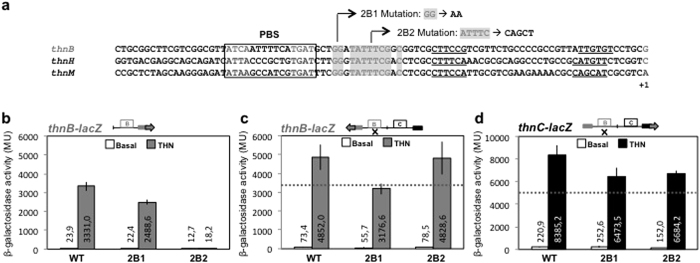
Mutations constructed at the SBS in the P_*B*_ region (**a**) and their effects on independent transcription from P_*C*_ (**b**) and on coordinated transcription from P_*B*_ (**c**) and P_*C*_ (**d**). The horizontal dotted lines represent the independent levels of expression.

Alignment of the P_*B*_, P_*H*_ and P_*M*_ promoter regulatory regions activated by ThnR (Fig. 6a) showed a similar conventional arrangement of the ThnR binding sites. They have a PBS centred at −64 relative to their transcriptional start site, followed by a second region that is conserved among them but very loosely related to the PBS, which may represent their ThnR SBS.

Two substitution mutants were created in this conserved region and the resulting mutant promoters used to construct *lacZ* gene fusions to analyse its relevance for independent or cooperative transcription from each promoter, as described above. The 2B1 mutation has the conserved GG dinucleotide at position −53 substituted by AA, whilst mutation 2B2 has the conserved ATTTC pentanucleotide at positions −49 to −45 substituted by CAGCT (Fig. 6a).

The 2B1 mutation had a weak reduction of independent transcription from P_*B*_ (75% of the wild-type levels) (Fig. 6b), which suggest that these nucleotides are relevant but not essential for *thnB* transcription. This mutation had a greater effect on P_*B*_ transcription in the complete context comprising both promoters (65% of the wild-type levels) (Fig. 6c), thus suggesting that this defect cannot be compensated by the presence of the *thnC* promoter region. The 2B1 mutation also affected the cooperative transcription from P_*C*_ although it did not abolish it as it retained 77% of the wild-type levels (Fig. 6d).

**Figure 7:**
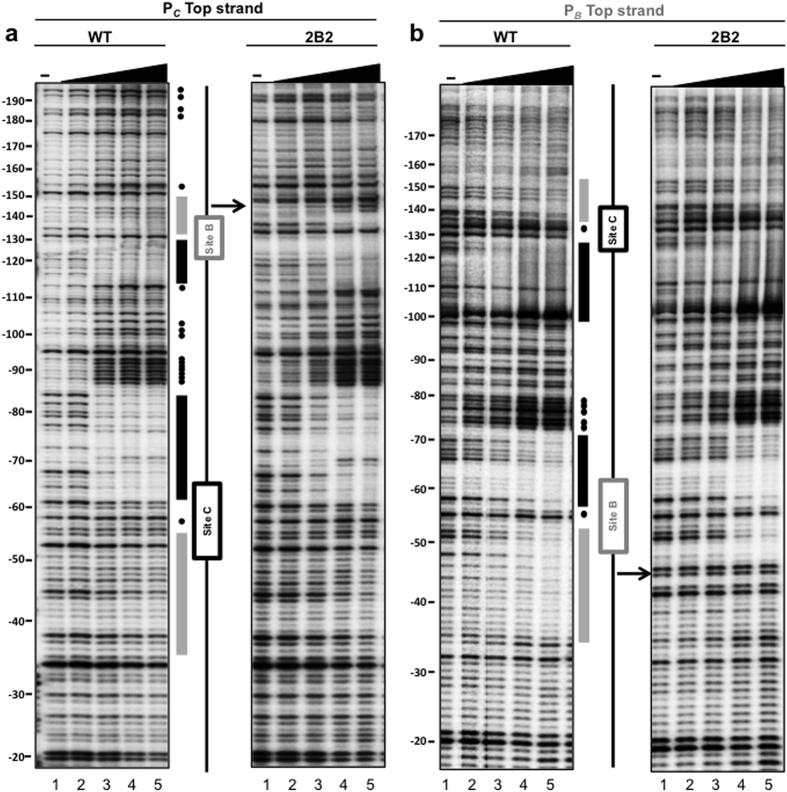
Footprints of WT and 2B2 substitution mutant. (**a**) P_*C*_ top strand and (**b**) P_*B*_ top strand. Black and grey rectangles represent protected regions at the primary and secondary binding sites of each promoter, respectively. Circles represent positions hypersensitive to DNase I treatment upon ThnR binding. Arrows represent the mutations location. The increasing concentrations of ThnR tetramers are: 0, 0.1, 0.5, 1 and 1.5 μM.

On the other hand, the pentanucleotide substitution in mutation 2B2 resulted in barely detectable levels of transcription activation from P_*B*_ in isolation (Fig. 6a), indicating that independent P_*B*_ transcriptional activation strictly requires productive interactions of ThnR with these sequences. Strikingly, in the presence of P_*C*_, the expression levels from the 2B2 mutant P_*B*_ were higher than those obtained from the 2B1 mutant and indistinguishable from those obtained with the wild-type region (Fig. 6c). These data indicates that the strong deficiency in the independent transcription activation caused by the 2B2 mutation can be fully compensated by the presence of the divergent P_*C*_ promoter region, thus making *thnB* expression in this context strictly dependent on cooperative transcription. Footprinting analysis of the divergent promoters region containing the 2B2 mutation showed that this secondary site B is not protected (Fig. 7), thus suggesting that productive interactions of ThnR with the conserved regions in the secondary site B are not essential for cooperative transcription from P_*B*_.

Finally, the 2B2 mutation had a weak effect, similar to 2B1 mutation, on transcription from P_*C*_ (Fig. 6d), thus suggesting that alteration of ThnR contacts in this region also affects but does not abolish cooperative transcription from P_*C*_.

### Role of the palindromic secondary site C in cooperative transcriptional activation

Deficiency in independent transcription from P_*B*_ provoked by 2B2 mutation was compensated by the presence of P_*C*_ promoter. In order to test the requirement of complex formation and the involvement of the secondary site C in this compensation, the 2B2 mutation was combined with the 1C mutation in the PBS, which is essential for P_*C*_ transcription and complex II formation^22^, with the 6 bp insertion that place the promoter regions on opposite faces of the helix, and with the 2C1 mutation in the P_*C*_ SBS, which showed a clear effect on the levels of cooperative transcription from both P_*C*_ and P_*B*_ (Fig. 5f,g), in the DNA fragment bearing both promoters.

**Figure 8:**
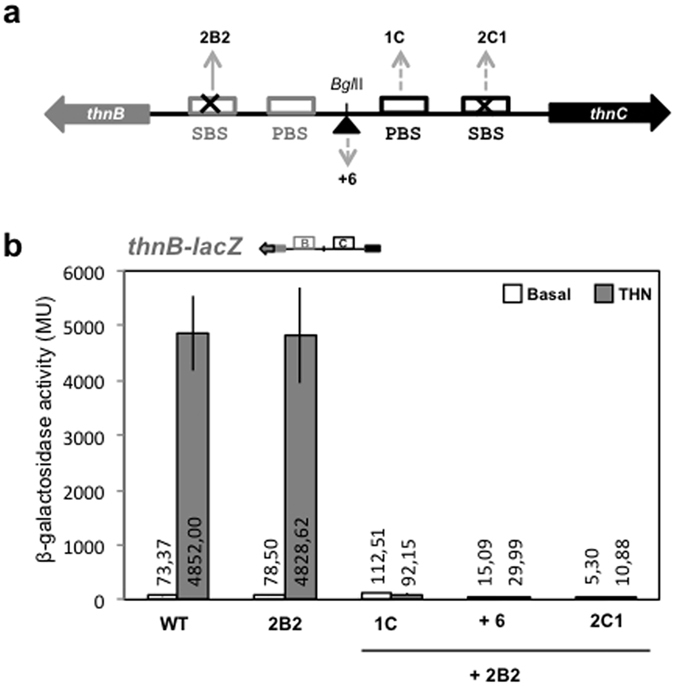
Effect of additional mutations in P_*C*_ on the strictly cooperative transcription from the 2B2 mutant P_*B*_ promoter. (**a**) Schematic representation of the mutations in the divergent promoters region. 1C is a mutation in the P_*C*_ PBS^22^ that virtually abolishes transcription from P_*C*_ in this system (not shown). (**b**) Expression levels from P_*B*_ when the 2B2 mutation is combined with others in the P_*C*_ promoter region.

As shown in Fig. 8, either insertion of half turn of the helix or the presence of the 1C or 2C1 mutations fully prevented transcription from the P_*B*_ promoter bearing the 2B2 mutation. This result clearly indicates that compensation of the deficiency provoked by the 2B2 mutation by the P_*C*_ promoter region strictly requires ThnR binding at its site in P_*C*_, complex formation and proper interactions of ThnR with its palindromic SBS at the divergent P_*C*_ promoter, thus establishing important elements of P_*C*_ for cooperative transcription from P_*B*_.

## Discussion

Regulation of the *thn* operons of *S. granuli* st. TFA by the activator ThnR is one of the increasing examples of non-conventional regulation by LysR-type regulators. The non-conventional features of this regulatory system are (i) the regulator ThnR does not repress its own transcription but actually activates it from the P_*C*_promoter^22^, (ii) ThnR requires the co-activator ThnY^24^, (iii) regulation directly affects not just one but at least four promoter targets promoter^21^, (iv) ThnR activates two closely linked divergent promoters, (v) activation of these divergent promoters is coordinated, and (vi) the primary binding site C is centred at −75 relative to the transcription start site^22^. Due to these differences, transcription activation, at least from the divergent promoters, deserves further characterisation, identification of the *cis-*acting sites and study of their relevance for independent and cooperative transcription.

Since a number of *in vivo* and *in vitro* experiments strongly support the existence of transcription-coupled DNA supercoiling at LTTR-regulated promoters^27^,^28^, we tested whether P_*C*_ − P_*B*_ cooperative expression was due to indirect effects caused by transcribing from the closely located divergent promoter. The mutation in the P_*C*_ −10 box, which severely reduced its functionality, had no effect on P_*B*_ transcription, indicating that P_*B*_ is not transcriptionally coupled to P_*C*_. On the other hand, the mutated −10 region of P_*B*_, which abolished transcription from P_*B*_, did only have a slight effect on P_*C*_ transcription. Moreover, transcriptional coupling cannot be the main mechanism for cooperative transcription from P_*C*_, since alteration of the phase of the helix, which should not affect transcriptional coupling via supercoiling, has a more drastic effect than that of the −10 box mutation in P_*B*_ (see below).

ThnR forms higher order structures as a result of protein-protein interactions when bound to their respective sites in both promoters^22^ (Fig. 4a). The complex is only formed when the primary binding sites B and C are aligned in the same face of the helix while tolerating separation of the binding sites by at least 3 integral turns of the helix, which indicates formation of a DNA loop in order to form the complex. The fact that expression from P_*C*_ is reduced in the insertion mutants that cannot form the complex but maintained in mutants with integral turns of the helix (Fig. 4c) rules out the possibility that a mechanism of transcriptional coupling via supercoiling is operating at P_*C*_, whilst supporting the view that cooperative transcription form P_*C*_ is achieved by formation of the ThnR complex via a DNA loop. However, a slight but significant decrease in P_*C*_ expression is observed in the P_*B*_ −10 box mutant (Fig. 3c). One possibility that reconcile these results is that cooperative transcription at P_*C*_ also requires incorporation of the RNA-polymerase at P_*B*_, which would modify the topology of the complex to facilitate transcriptional activation of P_*C*_.

On the other hand, cooperative transcription from P_*B*_ behaves differently because separation of the primary binding sites B and C results in a reduction of P_*B*_ expression, regardless their alignment in the same face of the helix (Fig. 4b), and this behaviour is not expected for a function that relies on protein-protein interaction from distant sites via formation of a loop. It is known that the intervening DNA in a DNA loop is not completely neutral and its alteration by imposing or relieving topological restrictions may affect functionality of the DNA loop^27^. However, these alterations affect complex formation or its stability that should be detected by EMSA, which is not the case in the intergenic *thnBC* region since insertion of 1 or 3 turns of the helix clearly allows complex formation (Fig. 4a). Therefore, complex formation may be required for cooperative transcription from P_*B*_ but it is not sufficient to guarantee it.

The presence of hypersensitive regions just upstream of each primary binding site indicates that ThnR distorts the DNA upon binding (Supplementary Fig. S1). These two distortions together with the ThnR protein-protein interactions can produce a complex in which ThnR bound to their respective sites fits within a rigid U-shaped cage with a precise topology strictly required for ThnR making productive contacts with the RNA-polymerase at P_*B*_. Increasing the distance between the binding sites may reduce rigidity to the U-shaped cage and the resulting topology of the complex would not be so precise, thus preventing cooperative transcription from P_*B*_.

Previous studies of this intergenic region showed that ThnR makes additional contacts downstream of the PBS sites thus extending towards the promoter^22^,^21^, but nothing was known about the importance of ThnR contacts with these regions. The mutation 2B2 in a conserved region in P_*B*_, P_*H*_ and P_*M*_, showed that this sequence was essential for independent transcription from P_*B*_. This region is located at the same position as the SBS sites in other LysR-regulated systems, relative to the −35/−10 promoter boxes, and also relative to the PBS. Therefore, although more mutations are required to better define the functional SBS required for activation, this conserved sequence represents an important part of the SBS at the P_*B*_ promoter.

On the other hand, a palindromic secondary site at P_*C*_ that is located at the same position as the so-called ABS sites in other systems relative to the −35/−10 promoter boxes, suggested that ThnR interaction with this site might be also important for productive contacts of ThnR with the RNA-polymerase at P_*C*_. However, mutations of both halves of this site showed that it is not important for independent transcription from P_*C*_; therefore, this palindromic SBS is not the functional equivalent of an ABS. On the contrary, mutation of the spacer region, just downstream of the primary binding site C, showed the essential role of this region for productive activation from P_*C*_. The spacer location relative to the PBS C is similar to that of the ABSs relative to their respective RBSs in other LysR-regulated systems; therefore, the spacer at the P_*C*_ promoter region is an SBS functionally equivalent to an ABS although its sequence does not resemble that of the SBS at P_*B*_, P_*H*_and P_*M*_.

There have been reports of functional LysR-type binding sites located at diverse positions relative to the transcription start sites of their activated promoters. However, in one example the promoter region had several LysR-type sites, one of which was in the conventional location^15^ while in the others, activation involved additional transcriptional activators that had a stronger effect than the LysR-type activator itself^16^, or the LysR-type regulator worked as an antirepressor^29^,^17^. However, the studies of the P_*C*_ regulatory region have shown that a LysR-type regulator may directly activate its target promoter when the relevant binding sites are located one turn of the helix upstream of the conserved position in the other LysR-regulated systems.

Strikingly, the 2B2 mutation, that almost completely impaired independent transcription from P_*B*_, had no effect at all in the whole context bearing both promoters, which indicates that formation of the complex at the divergent promoter may compensate deficient interactions of the activator with its binding site at one of the promoters. A number of advantages obtained by complex formation via DNA looping are conceivable^18^, the most obvious of them being minimisation of the mutations effects due to cooperative binding to suboptimal DNA binding sites^30^. However, this is not the case for the 2B2 mutation since binding affinity of ThnR to the P_*B*_ promoter region is dictated by its PBS (data not shown). The 2B2 mutation reveals an additional function of a DNA loop, which is to compensate deficiencies in the productive interaction of an activator with its binding site that allows precise positioning to achieve transcriptional activation. This compensation does not restore the original interaction of the activator with its SBS since the footprint showed no protection of the mutated sequence in 2B2 (Supplementary Fig. 4S). Rather, formation of the complex trough protein-protein interactions places ThnR in the right topological position to interact with the RNA-polymerase and activate P_*B*_ in spite of its deficient interaction with its SBS.

The arrangement of binding sites at P_*C*_ raises the question of why the sequences required for activation of P_*C*_ are one turn of the helix apart from the promoter and an apparently unnecessary palindromic SBS for ThnR appears between the relevant sites and the −35 promoter box. The palindromic site mutations 2C1 and 2C2 affected cooperative transcription from P_*C*_ and P_*B*_ even more than independent transcription from P_*C*_, thus suggesting that it might be involved in cooperative transcription. The inability to transcribe P_*B*_ when mutations 2B2 and 2C1 were combined more clearly revealed the role of the palindromic SBS in cooperative transcription activated by the complex. This unusual arrangement at the divergent promoters region also raises the question of how many ThnR tetramers are bound. The presence of the palindromic SBS at P_*C*_ opens the possibility that up to 3 ThnR tetramers could be simultaneously bound. Since interaction of ThnR with its SBS at P_*B*_ is not restored in the 2B2 mutants, the interesting possibility that the ThnR tetramer bound to the palindromic SBS at the P_*C*_ promoter region is actually responsible for activating P_*B*_in the 2B2 mutant cannot be ruled out. However, this activation mechanism requires formation of the whole complex including interaction of ThnR with an intact PBS at P_*B*_^22^.

LTTRs are the most abundant regulators of gene expression in bacteria and, although in most instances the activation mechanism appears to be simple and well conserved, in an increasing number of examples the activation process is more complex. In some cases, several sites for the LTTR are required at the promoter region^15^, while in others additional transcriptional activators are required for achieving maximal expression levels^19^,^31^,^16^,^32^,^8^. The direct role of the LTTR in activating transcription from these complex promoters is not clear and may be different because in some instances the LTTR appears to interact with the co-activator and play a direct role in activation^19^,^33^ whilst in others its role may be indirect by facilitating the action of other activators or working as an antirrepresson^17^. This paper describes a striking example of the diversity and complexity that can be observed in LTTR-mediated regulatory mechanisms, in which the activator, bound to one promoter region, may interact with itself when bound to another promoter region and influence its transcription at a distance by formation of a complex via a DNA-loop.

## Methods

### Media and growth conditions

*Escherichia coli* strains were grown in Luria–Beltrani medium (LB) at 37 °C. *S. granulis* strains were grown at 30 °C in MML-rich medium^34^ or minimal medium^35^ supplemented with 8 or 40 mM beta hydroxybutyrate as the carbon source.

### Plasmids, strains and oligonucleotides

Plasmids, bacterial strains and oligonucleotides are described in Supplementary Tables S1, S2 and S3, respectively.

P_*B*_ and P_*C*_ sequences were mutated by PCR, as previously described^36^ using the plasmid pIZ1001 as the template and the corresponding mutagenic primers (Supplementary Table S2). For mutations in the amplified DNA fragment was digested with BglII and ApaI and cloned in pIZ1002 and pIZ1003 by replacing the corresponding wild-type fragment. For mutations in P_*B*_ the amplified fragment was digested with BglII and EcoRI, and cloned in pIZ1002 and pIZ1003. For the double mutants, fragments containing the P_*C*_ single mutants were cloned in pMPO964 by replacing the corresponding fragment containing the wild-type P_*C*_ promoter.

To introduce one turn of the helix (11 bp) into the central position between site B and site C, the oligonucleotides thnB-11-thnC rv and thnB-11-thnC fwd were annealed and cloned into pIZ1002 digested with BglII. To introduce three turns of the helix (33 bp) into the central position between site B and site C, the oligonucleotides thnB-22-thnC rv and thnB-22-thnC fwd were annealed and cloned into pMPO918 (*thnC::lacZ* translational fusion) or pMPO919 (*thnB::lacZ* translational fusion) digested with EcoT22I. To introduce a half-turn of the helix (4 bp), pIZ1002 was digested with BglII, blunted with T4 DNA polymerase and re-ligated. To introduce a half-turn of the helix (6 bp), the thnB-6-thnC oligonucleotide was self-annealed and cloned into pIZ1002 digested with BglII. The resulting plasmid was digested at the newly created EcoT221 site, their ends made blunt with T4 DNA polymerase and re-ligated.

T690 is a strain lacking the *thnB* and *thnC* operons, which were deleted and substituted by a kanamycin-KIXX cassette from pUC4KIXX (Supplementary Table S2). Since the plasmids bearing the different *lacZ* gene fusions are not replicative in *S. granuli* In order to facilitate homologous recombination of these plasmids to integrate them into the T690 chromosome, the kanamycin-KIXX cassette from pUC4KIXX digested with EcoRI was cloned in the EcoRI site of these constructs.

Fragments containing all versions of the *thnB–thnC* intergenic region were amplified by PCR using the INT1- INT2 primers (introducing SalI and HindIII sites) and cloned in pBluescript II SK + digested with SalI and HindIII to generate the plasmids pMPO913, pMPO914, pMPO915, pMPO922, pMPO944, pMPO945, pMPO946, pMPO962, pMPO1539.

To monitor the expression levels of the *thn* genes in the mutant strain T690, plasmids bearing the different *thnB::lacZ* or *thnC::lacZ* gene fusions were introduced by electroporation and integrated by homologous recombination of the Km-KIXX cassettes present in the vector and in the chromosome of the T690 strain by selecting the ampicillin resistance encoded by the vectors.

### Induction assays

Induction assays with tetralin in the vapour phase were performed as previously described in minimal medium supplemented with 8 mM beta hydroxybutyrate^19^. The β-galactosidase activity of induced cultures of strains harbouring *lacZ* fusions integrated into the chromosome was assayed as described^37^.

Expression level values are the average of at least 9 independent biological replicas. Error bars in figures represent the standard deviation. Two-tailed Student t test was applied to compare values between the mutants and the wild-type promoter regions. All differences indicated in Results are statistically significant with a p value lower than 0.001.

### ThnR-His_6_ purification

A culture of the over-producing strain NCM631/pIZ227, pIZ1020 was grown overnight, diluted 100-fold in LB medium supplemented with ampicillin and chloramphenicol, and incubated at 37 °C to an OD_600_ of 0.3, cooled at 4 °C and 50 mM IPTG was added. The induction was carried out at 16 °C for 18 h. The cells corresponding to 8 litres of culture were chilled, harvested by centrifugation and lysed by sonication (12 min, 2 s on 2 s off, 40% amplitude) in 150 ml of chilled purification buffer (50 mM sodium phosphate, 400 mM KCl, 10 mM imidazole, 5% glycerol, 1 mM PMSF, pH 7.4). After sonication the extract was centrifuged 50 min at 90000 × g and 4 °C. Purification was performed by one-step affinity chromatography with Co^2+^ charged agarose beads as recommended by the manufacturer (High Density Cobalt 6BCL-QHCo-25, ABT). Imidazole (200 mM) was used to elute the protein, which was subsequently stored at −80 °C. Sample preparation and SDS-polyacrylamide electrophoresis to assess the overexpression and purification of ThnR-His_6_ were performed as previously described^38^. Gels were stained with the EZBlue stain reagent (Sigma). Protein concentrations were calculated using the Protein Assay Kit from Bio-Rad, according to the manufacturer’s protocol. Molarity of the protein samples was calculated assuming that ThnR is a tetramer.

### Electrophoretic mobility shift assays (EMSAs)

DNA probes for the *thnB–thnC* intergenic region (269 bp) containing both putative ThnR binding sites with different combinations of mutations in each site were obtained as *Sal*I–*EcoR*V fragments. The probes were [γ-^32^P]-dCTP labelled by Klenow filling of the 3′ recessed ends. EMSAs were performed as previously described^39^. ThnR–DNA complexes were generated in a 15 μl of reaction containing 2 fmol of each probe, 100 ng of salmon sperm DNA, 5 mg of BSA and increasing amounts of purified ThnR-His_6_. Gels were dried and exposed on a radiosensitive screen. Bands were visualized with a Typhoon 9410 scanner and analysed using the ImageQuant software (GE Healthcare)

### DNase I footprinting assays

DNA probes (269 bp) containing both B and C ThnR binding sites were prepared and labelled with ^32^P as described above. Footprint assays were performed essentially as previously described^39^. Binding reactions were performed with 2 fmol of the probe, 100 ng of salmon sperm DNA, 5 mg of BSA and increasing amounts of the purified ThnR-His_6_ in footprint buffer [10 mM Tris HCl (pH 8), 50 mM NaCl, 2 mM DTT, 10% glycerol, 1 mM CaCl_2_, 2 mM MgCl_2_]. A sequencing reaction performed with the Sequenase 2.0 kit (USB) using a specific oligonucleotide for the labelled strand was run in parallel as a size marker. The samples were run on a polyacrylamide sequencing gel. Dried gels were exposed to radiosensitive screens and scanned using a Typhoon 9410 scanner (GE Healthcare).

## Additional Information

**How to cite this article**: Rivas-Marín, E. *et al.* Genetic dissection of independent and cooperative transcriptional activation by the LysR-type activator ThnR at close divergent promoters. *Sci. Rep.* **6**, 24538; doi: 10.1038/srep24538 (2016).

## Supplementary Material

Supplementary Information
