# Supplementary material for: Genetic dissection of independent and cooperative transcriptional activation by the LysR-type activator ThnR at close divergent promoters
Source: Sci Rep. 2016 Apr 18;6:24538. doi: 10.1038/srep24538 (PMC4834489; doi:10.1038/srep24538)
Supplement: Supplementary Information [file srep24538-s1.pdf]

Genetic dissection of independent and cooperative transcriptional activation by the LysR-type activator ThnR at close divergent promoters.

Elena Rivas-Marín, Belén Floriano and Eduardo Santero

SUPPLEMENTARY INFORMATION

SUPPLEMENTARY FIGURES

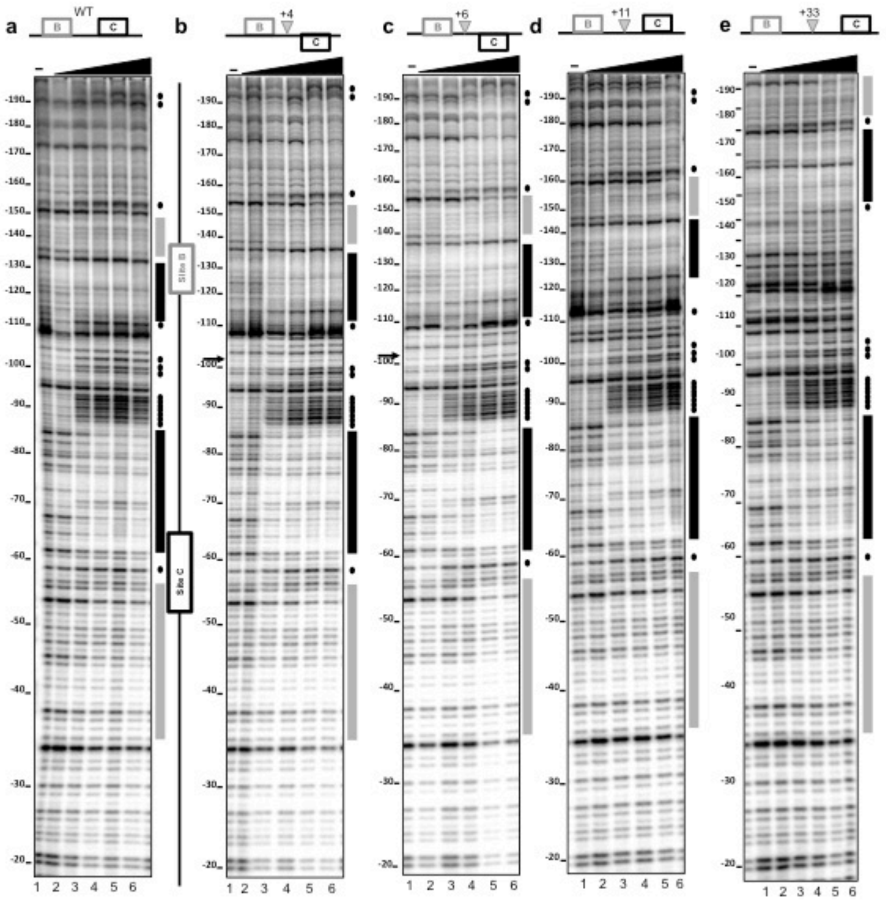

Supplementary Figure S1. Footprints of WT (a), +4 (b), +6 (c), +11 (d) and + 33 bp (e) insertion mutants in the intergenic P_B-P_C promoter region. Black and grey rectangles represent protected regions at the primary and secondary binding sites of each promoter, respectively. Circles represent positions hypersensitive to DNase I treatment upon ThnR binding. Arrows indicate the hypersensitive band missing in the outphased mutants but present in WT and +11 and +33 insertion mutants. The increasing concentrations of ThnR tetramers are: 0, 0.1, 0.5, 1, 1.5 and 1.8 μM.

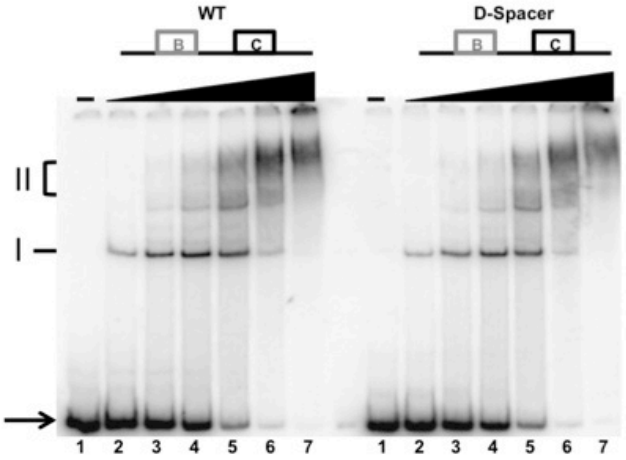

Supplementary Figure S2. Complex formation at the *thnB-thnC* divergent promoter region in WT and D-spacer mutant shown by EMSA. The increasing concentrations of ThnR tetramers are: 0, 10, 25, 50, 100, 200 and 400 nM.

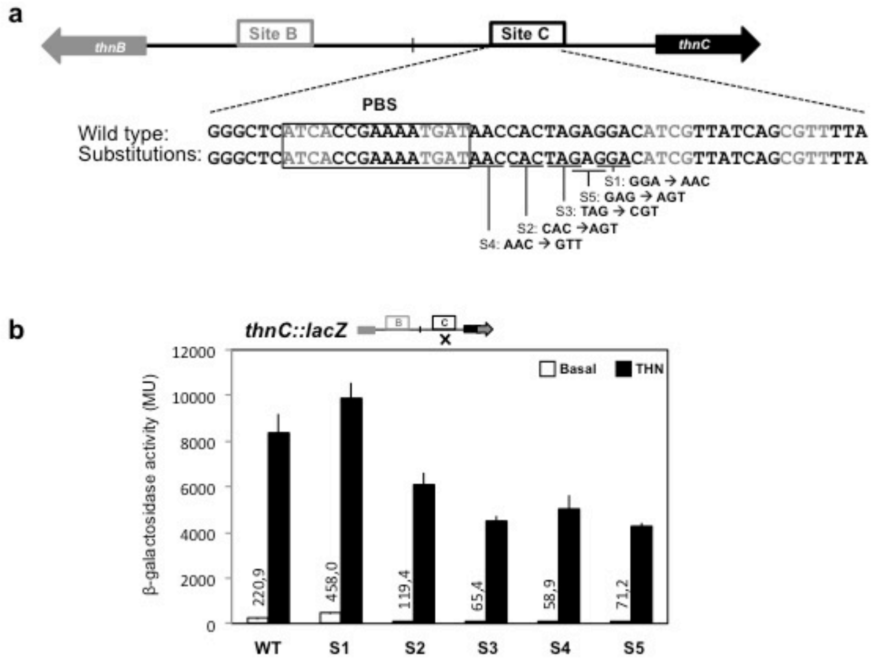

Supplementary Figure S3. S1 to S5 mutations constructed in the P_C spacer region (a) and their effect on P_C transcription (b).

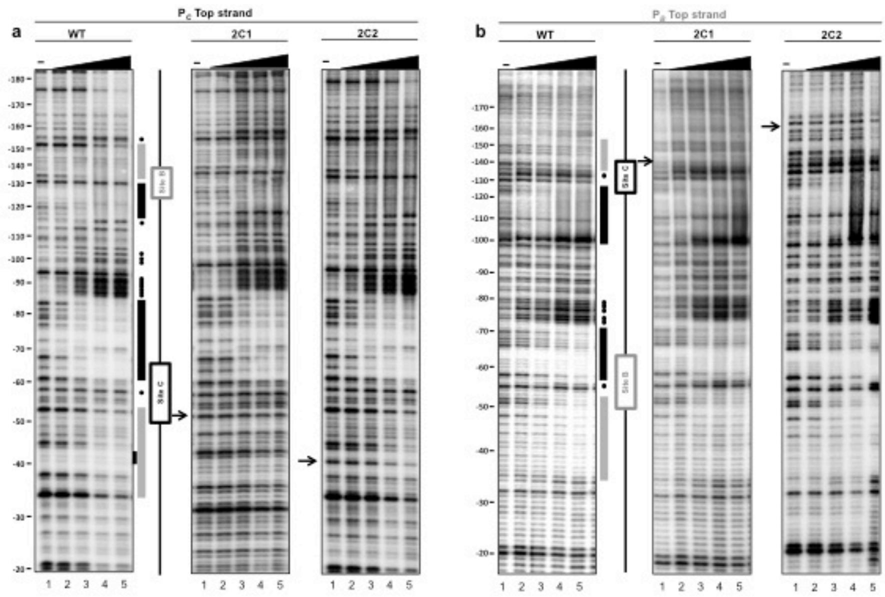

Supplementary Figure S4. Footprints of WT and mutants in the palindromic SBS of P$_C$. (a) P$_C$ top strand and (b) P$_B$ top strand. Black and grey rectangles represent protected regions at the primary and secondary binding sites of each promoter, respectively. Circles represent positions hypersensitive to DNase I treatment upon ThnR binding. Arrows represent the mutations location. The increasing concentrations of ThnR tetramers are: 0, 0.1, 0.5, 1 and 1.5 μM.

SUPPLEMENTARY TABLES

Supplementary Table S1. Plasmids used in this work.

Plasmids	Relevant characteristics	Reference
pBluescript II SK/KS+	Cloning vector. Ap^r.	Stratagene
pIZ227	LysE derivate, *lacI*^{q}. Cm^r.	40
pIZ1001	*thnB::thnC* intergenic region in pBluescript II SK$^+$. Ap^r.	19
pIZ1002	*thnC::lacZ* translational fusion bearing the whole *thnB–thnC* intergenic region. Ap^r.	19
pIZ1003	*thnB::lacZ* translational fusion bearing the whole *thnB–thnC* intergenic region. Ap^r.	19
pIZ1020	pT7-*thnR*,with six-histidine-tag fused in frame to C-terminus. AUG initiation codon. Ap^r.	22
pLAFR3	Broad host range plasmid. Tc^r.	41
pMPO513	269 bp fragment in pBluescript II SK+, bearing the wild-type *thnB–thnC* intergenic region. Ap^r.	This work
pMPO525	*thnC::lacZ* translational fusion, bearing the wild type P_C promoter region. Str^r, Km^r, Ap^r	22
pMPO526	*thnB::lacZ* translational fusion, bearing the wild type P_B promoter region. Str^r, Km^r, Ap^r	22
pMPO913	273 bp fragment in pBluescript II SK+, bearing the whole *thnB–thnC* intergenic region with a 4 bp insertion between B and C site. Ap^r.	This work
pMPO914	280 bp fragment in pBluescript II SK+, bearing the whole *thnB–thnC* intergenic region with a 11 bp insertion between B and C site. Ap^r.	This work
pMPO915	275 bp fragment in pBluescript II SK+, bearing the whole *thnB–thnC* intergenic region with a 6 bp insertion between B and C site. Ap^r.	This work
pMPO916	*thnC::lacZ* translational fusion, bearing the whole *thnB–thnC* intergenic region with a 4 bp insertion between B and C site. Km^r, Ap^r.	This work
pMPO917	*thnB::lacZ* translational fusion, bearing the whole *thnB–thnC* intergenic region with a 4 bp insertion between B and C site. Km^r, Ap^r.	This work
pMPO918	*thnC::lacZ* translational fusion, bearing the whole *thnB–thnC* intergenic region with an 11 bp insertion between B and C site. Km^r, Ap^r.	This work
pMPO919	*thnB::lacZ* translational fusion, bearing the whole *thnB–thnC* intergenic region with an 11 bp insertion between B and C site. Km^r, Ap^r.	This work
pMPO920	*thnC::lacZ* translational fusion, bearing the whole *thnB–thnC* intergenic region with a 6 bp insertion between B and C site. Km^r, Ap^r.	This work
pMPO921	*thnB::lacZ* translational fusion, bearing the whole *thnB–thnC* intergenic region with a 6 bp insertion between B and C site. Km^r, Ap^r.	This work
pMPO922	302 bp fragment in pBluescript II SK+, bearing the whole *thnB–thnC* intergenic region with a 33 bp insertion between B and C site. Ap^r.	This work
pMPO923	*thnC::lacZ* translational fusion, bearing the whole *thnB–thnC* intergenic region	This work

	with a 33 bp insertion between B and C site. Km^r, Ap^r.	
pMPO924	*thnB::lacZ* translational fusion, bearing the whole *thnB–thnC* intergenic region with a 33 bp insertion between B and C site. Km^r, Ap^r.	This work
pMPO928	*thnC::lacZ* translational fusion, bearing the whole *thnB–thnC* intergenic region with -10 box of P_C mutated. Km^r Ap^r.	This work
pMPO929	*thnB::lacZ* translational fusion, bearing the whole *thnB–thnC* intergenic region with -10 box of P_C mutated. Km^r Ap^r.	This work
pMPO943	*thnA4´RY* cloned in pLAFR3. Tc^r.	This work
pMPO944	269 bp fragment in pBluescript II SK+, bearing the whole *thnB–thnC* intergenic region with 2B1 mutation in P_B. Ap^r.	This work
pMPO945	269 bp fragment in pBluescript II SK+, bearing the whole *thnB–thnC* intergenic region with 2C1 mutation in P_C. Ap^r.	This work
pMPO946	269 bp fragment in pBluescript II SK+, bearing the whole *thnB–thnC* intergenic region with a 12 bp deletion in P_C. Ap^r.	This work
pMPO947	*thnC::lacZ* translational fusion, bearing the whole *thnB–thnC* intergenic region with 2B1 mutation in P_B. Km^r Ap^r.	This work
pMPO948	*thnB::lacZ* translational fusion, bearing the whole *thnB–thnC* intergenic region with 2B1 mutation in P_B. Km^r Ap^r.	This work
pMPO949	*thnC::lacZ* translational fusion, bearing the whole *thnB–thnC* intergenic region with 2C1 mutation in P_C Km^r Ap^r.	This work
pMPO950	*thnB::lacZ* translational fusion, bearing the whole *thnB–thnC* intergenic region with 2C1 mutation in P_C. Km^r Ap^r.	This work
pMPO951	*thnC::lacZ* translational fusion, bearing the whole *thnB–thnC* intergenic region with a 12 bp deletion in P_C. Km^r Ap^r.	This work
pMPO952	*thnB::lacZ* translational fusion, bearing the whole *thnB–thnC* intergenic region with a 12 bp deletion in P_C. Km^r Ap^r.	This work
pMPO962	269 bp fragment in pBluescript II SK+, bearing the whole *thnB–thnC* intergenic region with 2B2 mutation in P_B. Ap^r.	This work
pMPO963	*thnC::lacZ* translational fusion, bearing the whole *thnB–thnC* intergenic region with 2B2 mutation in P_B. Km^r Ap^r.	This work
pMPO964	*thnB::lacZ* translational fusion, bearing the whole *thnB–thnC* intergenic region with 2B2 mutation in P_B. Km^r Ap^r.	This work
pMPO966	*thnC::lacZ* translational fusion, bearing the whole *thnB–thnC* intergenic region with -10 box of P_B mutated. Km^r Ap^r.	This work
pMPO967	*thnB::lacZ* translational fusion, bearing the whole *thnB–thnC* intergenic region with -10 box of P_B mutated. Str^r Km^r Ap^r.	This work
pMPO968	*thnB::lacZ* translational fusion, bearing the P_B promoter region with 2B1 mutation. Km^r Ap^r.	This work
pMPO969	*thnC::lacZ* translational fusion, bearing the P_C promoter region with 2C1 mutation. Km^r Ap^r.	This work
pMPO970	*thnC::lacZ* translational fusion, bearing the P_C promoter region with a 12 bp deletion. Km^r Ap^r.	This work
pMPO971	*thnB::lacZ* translational fusion, bearing the P_B promoter region with the 2B2	This work

	mutation. Km^r Ap^r.	
pMPO978	*thnB::lacZ* translational fusion, bearing the whole *thnB–thnC* intergenic region with the 2C2 mutation in P_C, and the 2B2 mutation in P_B. Km^r Ap^r.	This work
pMPO985	*thnC::lacZ* translational fusion, bearing the whole *thnB–thnC* intergenic region with a 12 bp substitution in P_C. Km^r Ap^r.	This work
pMPO986	*thnC::lacZ* translational fusion, bearing the P_C promoter region with a 12 bp substitution. Km^r Ap^r.	This work
pMPO1512	*thnC::lacZ* translational fusion, bearing the whole *thnB–thnC* intergenic region with a 3 bp substitution (1) in P_C. Km^r Ap^r.	This work
pMPO1521	*thnC::lacZ* translational fusion, bearing the whole *thnB–thnC* intergenic region with a 3 bp substitution (2) in P_C. Km^r Ap^r.	This work
pMPO1522	*thnC::lacZ* translational fusion, bearing the whole *thnB–thnC* intergenic region with a 3 bp substitution (3) in P_C. Km^r Ap^r.	This work
pMPO1525	*thnC::lacZ* translational fusion, bearing the whole *thnB–thnC* intergenic region with a 6 bp insertion between B and C site, and the 2B2 mutation in P_B. Km^r Ap^r.	This work
pMPO1526	*thnB::lacZ* translational fusion, bearing the whole *thnB–thnC* intergenic region with a 6 bp insertion between B and C site, and the 2B2 mutation in P_B. Km^r Ap^r.	This work
pMPO1529	*thnC::lacZ* translational fusion, bearing the whole *thnB–thnC* intergenic region with a 3 bp substitution (4) in P_C. Km^r Ap^r.	This work
pMPO1530	*thnC::lacZ* translational fusion, bearing the whole *thnB–thnC* intergenic region with a 3 bp substitution (5) in P_C. Km^r Ap^r.	This work
pMPO1534	*thnC::lacZ* translational fusion, bearing the whole *thnB–thnC* intergenic region with 2C2 mutation in P_C. Km^r Ap^r.	This work
pMPO1535	*thnB::lacZ* translational fusion, bearing the whole *thnB–thnC* intergenic region with 2C2 mutation in P_C. Km^r Ap^r.	This work
pMPO1538	*thnC::lacZ* translational fusion, bearing the P_C promoter region with 2C2 mutation. Km^r Ap^r.	This work
pMPO1539	269 bp fragment in pBluescript II SK+, bearing the whole *thnB–thnC* intergenic region with 2B2 mutation in P_B. Ap^r.	This work
pMPO1543	*thnB::lacZ* translational fusion, bearing the whole *thnB–thnC* intergenic region with 2C1 mutation in P_C, and 2B2 mutation in P_B. Km^r Ap^r.	This work
pT7-7	Expression vector bearing the ϕ10 gene promoter and ribosome binding site. Ap^r.	42
pUC4-KIXX	Kanamycin resistance gene KIXX cassette from Tn5 in pUC4K. Ap^r, Km^r.	Pharmacia

Supplementary Table S2. Bacterial strains used in this work.

Strains	Relevant characteristics	Reference
E. coli		
DH5α	F- ϕ80*lacZ*ΔM15 Δ(*lacZYA-argF)U169 recA1 endA1 hsdR17*(rK-m K-) *supE44 thi-1 gyrA relA1*	43
NCM631	*HsdS gal lacZ*ΔM15 lacI *lacUV5:gen1*(T7 RNA polymerase) *Δlac*-Tn10	40
S. granulis		
TFA	Wild type. Str^r	44
T690	*Δthn*. 12,2 Kb of the genome covering the whole *thnC* operon and the *thnB* operon down to *thnC* substituted by a KIXX insertion. Str^r, Km^r.	45
T690:525	*Δthn, thnC::lacZ* translational fusion, bearing the wild type P_C promoter region. Str^r, Km^r, Ap^r.	22
T690:526	*Δthn, thnB::lacZ* translational fusion, bearing the wild type P_B promoter region. Str^r, Km^r, Ap^r.	
T690:690	*Δthn, thnC::lacZ* translational fusion, wild type *thnB-thnC* intergenic region. Str^r, Km^r, Ap^r.	22
T690:693	*Δthn, thnB::lacZ* translational fusion, wild type *thnB-thnC* intergenic region. Str^r, Km^r, Ap^r.	22
T690:916	*Δthn, thnC::lacZ* translational fusion, bearing the whole *thnB–thnC* intergenic region with a 4 bp insertion between B and C site. Str^r, Km^r, Ap^r.	This work
T690:917	*Δthn, thnB::lacZ* translational fusion, bearing the whole *thnB–thnC* intergenic region with a 4 bp insertion between B and C site. Str^r, Km^r, Ap^r.	This work
T690:918	*Δthn, thnC::lacZ* translational fusion, bearing the whole *thnB–thnC* intergenic region with an 11 bp insertion between B and C site. Str^r, Km^r, Ap^r.	This work
T690:919	*Δthn, thnB::lacZ* translational fusion, bearing the whole *thnB–thnC* intergenic region with an 11 bp insertion between B and C site. Str^r, Km^r, Ap^r.	This work
T690:920	*Δthn, thnC::lacZ* translational fusion, bearing the whole *thnB–thnC* intergenic region with a 6 bp insertion between B and C site. Str^r, Km^r, Ap^r.	This work
T690:921	*Δthn, thnB::lacZ* translational fusion, bearing the whole *thnB–thnC* intergenic region with a 6 bp insertion between B and C site. Str^r, Km^r, Ap^r.	This work
T690:923	*Δthn, thnC::lacZ* translational fusion, bearing the whole *thnB–thnC* intergenic region with a 33 bp insertion between B and C site. Str^r, Km^r, Ap^r.	This work
T690:924	*Δthn, thnB::lacZ* translational fusion, bearing the whole *thnB–thnC* intergenic region with a 33 bp insertion between B and C site. Str^r, Km^r, Ap^r.	This work
T690:928	*Δthn, thnC::lacZ* translational fusion, bearing the whole *thnB–thnC* intergenic region with the -10 box of P_C mutated. Str^r Km^r Ap^r.	This work
T690:929	*Δthn, thnB::lacZ* translational fusion, bearing the whole *thnB–thnC* intergenic region with the -10 box of P_C mutated. Str^r Km^r Ap^r.	This work
T690:947	*Δthn, thnC::lacZ* translational fusion, bearing the whole *thnB–thnC* intergenic region with the 2B1 mutation in P_B. Str^r Km^r Ap^r.	This work
T690:948	*Δthn, thnB::lacZ* translational fusion, bearing the whole *thnB–thnC* intergenic region with the 2B1 mutation in P_B. Str^r Km^r Ap^r.	This work
T690:949	*Δthn, thnC::lacZ* translational fusion, bearing the whole *thnB–thnC* intergenic	This work

	region with the 2C1 mutation in P_C. Str^r Km^r Ap^r.	
T690:950	*Δthn, thnB::lacZ* translational fusion, bearing the whole *thnB–thnC* intergenic region with the 2C1 mutation in P_C. Str^r Km^r Ap^r.	This work
T690:951	*Δthn, thnC::lacZ* translational fusion, bearing the whole *thnB–thnC* intergenic region with a 12 bp deletion in P_C. Str^r Km^r Ap^r.	This work
T690:952	*Δthn, thnB::lacZ* translational fusion, bearing the whole *thnB–thnC* intergenic region with a 12 bp deletion in P_C. Str^r Km^r Ap^r.	This work
T690:963	*Δthn, thnC::lacZ* translational fusion, bearing the whole *thnB–thnC* intergenic region with the 2B2 mutation in P_B. Str^r Km^r Ap^r.	This work
T690:964	*Δthn, thnB::lacZ* translational fusion, bearing the whole *thnB–thnC* intergenic region with the 2B2 mutation in P_B. Str^r Km^r Ap^r.	This work
T690:966	*Δthn, thnC::lacZ* translational fusion, bearing the whole *thnB–thnC* intergenic region with the -10 box of P_B mutated. Str^r Km^r Ap^r.	This work
T690:967	*Δthn, thnB::lacZ* translational fusion, bearing the whole *thnB–thnC* intergenic region with the -10 box of P_B mutated. Str^r Km^r Ap^r.	This work
T690:968	*Δthn, thnB::lacZ* translational fusion, bearing the P_B promoter region with the 2B1 mutation. Str^r Km^r Ap^r.	This work
T690:969	*Δthn, thnC::lacZ* translational fusion, bearing the P_C promoter region with the 2C1 mutation. Str^r Km^r Ap^r.	This work
T690:970	*Δthn, thnC::lacZ* translational fusion, bearing the P_C promoter region with a 12 bp deletion. Str^r Km^r Ap^r	This work
T690:971	*Δthn, thnB::lacZ* translational fusion, bearing P_B promoter region with the 2B2 mutation. Str^r Km^r Ap^r.	This work
T690:978	*Δthn, thnB::lacZ* translational fusion, bearing the whole *thnB–thnC* intergenic region with the 2C2 mutation in P_C, and the 2B2 mutation in P_B. Str^r Km^r Ap^r.	This work
T690:985	*Δthn, thnC::lacZ* translational fusion, bearing the whole *thnB–thnC* intergenic region with a 12 bp substitution in P_C. Str^r Km^r Ap^r.	This work
T690:986	*Δthn, thnC::lacZ* translational fusion, bearing the P_C promoter region with a 12 bp substitution. Str^r Km^r Ap^r	This work
T690:1512	*Δthn, thnC::lacZ* translational fusion, bearing the whole *thnB–thnC* intergenic region with a 3 bp substitution (1) in P_C. Str^r Km^r Ap^r.	This work
T690:1521	*Δthn, thnC::lacZ* translational fusion, bearing the whole *thnB–thnC* intergenic region with a 3 bp substitution (2) in P_C. Str^r Km^r Ap^r.	This work
T690:1522	*Δthn, thnC::lacZ* translational fusion, bearing the whole *thnB–thnC* intergenic region with a 3 bp substitution (3) in P_C. Str^r Km^r Ap^r.	This work
T690:1525	*Δthn, thnC::lacZ* translational fusion, bearing the whole *thnB–thnC* intergenic region with a 6 bp insertion between B and C site, and the 2B2 mutation in P_B. Str^r Km^r Ap^r.	This work
T690:1526	*Δthn, thnB::lacZ* translational fusion, bearing the whole *thnB–thnC* intergenic region with a 6 bp insertion between B and C site, and the 2B2 mutation in P_B. Str^r Km^r Ap^r.	This work
T690:1529	*Δthn, thnC::lacZ* translational fusion, bearing the whole *thnB–thnC* intergenic region with a 3 bp substitution (4) in P_C. Str^r Km^r Ap^r.	This work

T690:1530	*Δthn, thnC::lacZ* translational fusion, bearing the whole *thnB–thnC* intergenic region with a 3 bp substitution (5) in P_C. Str^r Km^r Ap^r.	This work
T690:1534	*Δthn, thnC::lacZ* translational fusion, bearing the whole *thnB–thnC* intergenic region with the 2C2 mutation in P_C. Str^r Km^r Ap^r.	This work
T690:1535	*Δthn, thnB::lacZ* translational fusion, bearing the whole *thnB–thnC* intergenic region with the 2C2 mutation in P_C. Str^r Km^r Ap^r.	This work
T690:1538	*Δthn, thnC::lacZ* translational fusion, bearing the P_C promoter region with the 2C2 mutation. Str^r Km^r Ap^r.	This work
T690:1543	*Δthn, thnB::lacZ* translational fusion, bearing the whole *thnB–thnC* intergenic region with the 2C1 mutation in P_C, and the 2B2 mutation in P_B. Str^r Km^r Ap^r.	This work

Supplementary Table S3. Oligonucleotides used in this work. The names of the oligonucleotides indicate the mutations they produce when used as the mutagenic primers.

Primers	Sequence 5´-> 3	Reference
INT1	TTGCCAGTCGACGGTTCGCGCCCGAAAATTC	22
INT2	CTCGCGAAGCTTCGCTGATGCGGTTAC	22
-10 P_C mutation fw	TCATTCCGCATGACGTGCACACCAGG	This work
-10 P_C mutation rv	CCTGGTGTGCACGTCATGCGGAATGA	This work
-10 P_B mutation fw	ATCGCAGGACCACCGCACGGCGGGGC	This work
-10 P_B mutation rv	GCCCCGCCGTGCGGTGGTCCTGCGAT	This work
+ 6 bp insertion	GATCATGCAT	This work
+11 bp insertion fw	GATCATGCATC	This work
+11 bp insertion rv	GATCGATGCAT	This work
+33 bp insertion fw	GACGTCATTCAGTACTAGTGCA	This work
+33 bp insertion rv	CTAGTACTGAATGACGTCTGCA	This work
2B1 mutation rv	GCCGAAATATAAAGCATCATG	This work
2B1 mutation fw	CATGATGCTTTATATTTCGGC	This work
2C1 mutation fw	CTAGAGGACAAAGTTATCAGCG	This work
2C1 mutation rv	CGCTGATAACTTTGTCCTCTA	This work
D-spacer mutation fw	CTCATCACCGAAAATGATACATCGTTATCAGCGTTTTAC	This work
D-spacer mutation rv	GTAAAACGCTGATAACGATGTATCATTTTCGGTGATGAG	This work
S-spacer mutation fw	CATCACCGAAAATGATGGTTGTCGAGAAACATCGTTATCAGCG	This work
S-spacer mutation rv	CGCTGATAACGATGTTTCTCGACAACCATCATTTTCGGTGATG	This work
2C2 mutation fw	TCGTTATCAGCCGGTTACTGCGCC	This work
2C2 mutation rv	GGCGCAGTAACCGGCTGATAACG	This work
2B2 mutation fw	GGATCAGCTGGCGGTCGCTTCCGTCGTTC	This work
2B2 mutation rv	GCCAGCTGATCCAGCATCATGAAAATTGATAAC	This work
3bp substitution 1 fw	ATAACCACTAGAAACCATCG	This work
3bp substitution 1 rv	CGATGGTTTCTAGTGGTTATC	This work

3bp substitution 2 fw	AAATGATAACAGTTAGAGGACATC	This work
3bp substitution 2 rv	ATGTCCTCTAACTGTTATCATTTTC	This work
3bp substitution 3 fw	TGATAACCACCGTAGGACATCG	This work
3bp substitution 3 rv	CGATGTCCTACGGTGGTTATC	This work
3bp substitution 4 fw	CGAAAATGATGTTCACTAGAGGAC	This work
3bp substitution 4 rv	GTCCTCTAGTGAACATCATTTTCG	This work
3bp substitution 5 fw	GATAACCACTAAGTGACATCGTTATC	This work
3bp substitution 5 rv	GATAACGATGTCACTTAGTGGTTATC	This work

SUPPLEMENTARY REFERENCES

40. Govantes, F. & Santero, E. Transcription termination within the regulatory *nifLA* operon of *Klebsiella pneumoniae*. *Mol. Gen. Genet.* **250**, 447-54 (1996).

41. Staskawicz, B., Dahlbeck, D., Keen, N. & Napoli, C. Molecular characterization of cloned avirulence genes from race 0 and race 1 of *Pseudomonas syringae* pv. glycinea. *J. Bateriol.* **169**, 5789-94 (1987).

42. Tabor, S. & Richardson, C.C. A bacteriophage T7 RNA polymerase/promoter system for controlled exclusive expression of specific genes. *Proc. Natl. Acad. Sci. USA*. **82**, 1074-8 (1985).

43. Hanahan, D. Studies on transformation of *Escherichia coli* with plasmids. *J. Mol Biol.* **166**, 557-80 (1983).

44. Hernáez, M.J., Reineke, W. & Santero, E. Genetic analysis of biodegradation of tetralin by a *Sphingomonas* strain. *Appl. Environ. Microbiol.* **65**, 1806-10 (1999).

45. Moreno-Ruíz, E., Hernáez, M.J., Martínez-Pérez, O. & Santero, E. Identification and functional characterization of *Sphingomonas macrogolitabida* strain TFA genes involved in the first two steps of the tetralin catabolic pathway. *J. Bacteriol.* **185**, 2026-30 (2003).
